# Automobile Hints

**Published:** 1918-01

**Authors:** 


### AUTOMOBILE HINTS.

**The Beginner** should realize that the care of an automobile involves a great deal of hard work, about half an hour labor to every hour of running. A man who is liable to injury from strain and exposure to cold after overheating, who has a hernia liable to become incarcerated, a chronic nephritis, brittle arteries, a bad heart or any comparable lesion, should not have an automobile unless he can have some one run it for him, all the time.

**Before Buying a New Car,** especially an orphan, get the actual experience of several purchasers who have run it for 5,000 miles or more, as to mileage per gallon of gasoline and oil, whether it is too heavy for the tires, whether it stands up well and whether it is convenient to care for. Trivial but really important points are whether there is space for carrying oil, extra tires, etc., whether the engine oil receptacle can be filled without special funnels, whether the greasing places are unreasonably numerous and of different kinds, whether they are accessible, how often they need attention, and whether grease cups, etc., are tight and properly threaded. (We speak from current unfortunate experience). For instance, in an average yearly run of 10,000 miles, it makes a difference of a full day's time, whether greasings have to be done every 50 or every 200 miles, whether you have to dismember the car to give it routine attention, whether a few, tight, properly placed greasing points can be attended to in five minutes, or whether you have to dress for the occasion in overalls and waste ten or fifteen minutes because a grease cup has come off or sticks or the thread allows only a few turns or does not catch at all.

**Winter Care.** Drain the radiator and have rubber connections replaced if worn and even small leaks repaired. It makes a difference whether you are wasting water or alcohol. As you drain the radiator, collect the water and measure carefully to know the contents of the radiation system. It is, of course, more convenient to have this in liters and c.c. than gallons and quarts. A gallon is almost exactly 3750 c.c. Denatured and wood alcohol are about equally good and are the only practical anti-freeze substances. Glycerine, 5-10% is not so much a protection against freezing as a preventive of the more rapid evaporation of the alcohol. It is expensive and is not absolutely efficient for the purpose stated. The simplest method is to fill the radiator with either kind of alcohol, 33 1/3% in water, which will protect down to about 15° below zero Fahrenheit. Such a mixture, however, boils when running at moderate winter temperatures, especially with a hood cover. It is, therefore, unsuited for the majority of winter demands and it tends to lose its alcohol rapidly, so that it is inefficient against freezing when the cold becomes extreme. A good rule is that every 5% of alcohol protects for 7° F. below freezing point. Hence, as cold weather begins, add 5% of the total contents of the radiator, having purposely kept the water rather lower than in summer, to allow room. Always add alcohol before running the engine, to provide for thorough mixture, and preferably after standing long enough for the contents to cool, to avoid considerable immediate evaporation. When the temperature is likely to go below 25° F., add 5% more, protecting down to 18° and so on. 25% protects to—3° F., theoretically. Practically, the alcohol of either kind, as purchased, is not quite 100% strength and, even if glycerine is used, the alcohol will evaporate faster than the water. On the other hand, if a hood cover is used and the car is not kept standing in the cold for more than 3-6 hours, according to the capacity of the radiator, only a little of the lower part of the radiator will freeze and then in the form of only slightly expansive slush-ice, so that the walls are not apt to be ruptured. By keeping a record of the actual amounts of alcohol added from time to time and adding them, the percentage can be readily calculated, except that allowance must be made as just indicated. On reaching the minimum temperature likely to be encountered, it is well to make up a solution of the corresponding strength (15% for 11° F., 20% for 4° F., 25% for 3° F.) and add this as required, until the cold abates when weaker solutions can be used and finally plain water. When danger of freezing is past, drain and wash the radiator and start on plain water again. A little extra alcohol should be added occasionally to allow for excess evaporation. When you put the car up on an unusually cold night, siphon off or drain out a small vial of the radiator mixture and set it outdoors to note whether it freezes and whether, if so, it is frozen solid or only with a little slush at the bottom. This simple test will show whether you are protected or not and you can even refine the test by taking the critical point with a laboratory thermometer and calculating the percentage actually present by the rule already given. Remember that if you have had a mechanic do anything to your car beyond tightening a nut on the chassis, he has probably drained the radiator, thrown away or kept the alcohol mixture and filled the radiator with plain water.

**Starting in Winter.** If you use a storage battery, have it tested at the beginning of cold weather. Batteries freeze. A hood cover probably pays for itself in gasoline and battery wear, in the course of a winter. Drain out old engine oil, clean spark plugs, brighten and tighten all ignition connections, have the coil points filed and adjusted and use a slightly richer gas mixture by permanent adjustment of the carburetor. In other words, remove all possible obstacles to ignition. Remember that a self starter is simply a substitute for hand cranking. For moderate degrees of cold of the engine itself—depending, of course, not only on out-door temperature but the time the car has stood without running—it is necessary only to enrich the mixture and to choke off the air admitted. For very cold engines, actual priming, as by a cup leading to the manifold for machines not provided with funnels and pet cocks, or the use of 2 c.c. of gasoline poured into two or more cylinders through the funnels, should be practiced. With these precautions, 6 quarter turns for a Ford or two or three brief spins with a starter on other cars should suffice even after long standing on a cold night. When you hear a starter going for 10 or 15 minutes it means one of two things, either the man in the car is richer or less rational than his mixture.

**Tires.** Pneumonia has followed the chill due to perspiration in changing a tire. If possible start the winter with new tires. Use easily detachable chains. Carry inflated tires on wheels or rims that are really easily applied. If these precautions are not feasible, risk spoiling a tire by running flat to the nearest garage. A short run in snow does comparatively little harm as a rule.

**Accidental Ignition.** A man in Dubuque, last winter, developed enough frictional electricity by the movement of his arms while walking in a fur coat, and this was stored by the fact that he was insulated by rubber boots so that, when he used a priming cup, it exploded. The man was badly burned and the car and garage completely destroyed.

**Exhaust Gases.** We have already called attention to a fatal case in Buffalo. Never let the engine run for more than a minute or two in a closed garage. If necessary to make tests with the engine running, open the door or fasten a piece of garden hose over the exhaust pipe and conduct the gases outdoors through an augur hole.

**Safety First.** The peculiar conditions due to mud, wet pavements, especially before oil has been washed away, dead leaves, snow and ice, can be determined only by actual experience. This experience may not be of further use unless gradually acquird at slow speed. As a rough rule, the following speeds may be considered safe: No danger of skidding, brakes in good condition, vision clear, and unobstructed, 10 miles an hour at corners and crossings, 15 in the middle of blocks in a city or village, 20 in long city blocks with little traffic, 30 in the country, with no vehicles within 500 feet. If any of the favorable factors are wanting, the limits are 5-20 miles. Most accidents to pedestrians and a majority to automobilists are due to insistence on right of way. Always give the right of way to a child, a delivery Ford, a truck and a dealer. If, for any reason you can not see a clear road ahead for 500 feet, blow the horn, keep well to the right and go into neutral or reduce speed.

